# Poisoning from Aniline Dyes

**Published:** 1914-02

**Authors:** 


POISONING FROM ANILINE DYES. Creyz, *Jour. de Med. de Bordeaux,* August 3, 1913, reports two cases of syncope, one with cyanosis and melanuria, due to dying tan shoes with aniline black, and a similar case due to aniline green in stockings. In the latter case, the symptoms came on after wearing the stockings for about six hours, when the patient, who believed that her dizziness was an indication of pregnancy, would go to bed and recover. Several attacks occurred before pregnancy was disproved and the stockings destroyed.

